# Transcatheter aortic valve implantation vs. surgical aortic valve replacement for aortic stenosis in Taiwan: A population-based cohort study

**DOI:** 10.1371/journal.pone.0285191

**Published:** 2023-05-03

**Authors:** Ching-Hu Chung, Yu-Jen Wang, Xiayu Jiao, Chia-Ying Lee

**Affiliations:** 1 Department of Medicine, Mackay Medical College, New Taipei City, Taiwan; 2 Edwards Lifesciences (Taiwan) Corp, Taipei, Taiwan; 3 Edwards Lifesciences, Irvine, CA, United States of America; Taipei Medical University, TAIWAN

## Abstract

**Objective:**

Aortic stenosis (AS) is a heart valve disease characterized by left ventricular outflow fixed obstruction. It can be managed by surgical aortic valve replacement (SAVR) or transcatheter aortic valve implantation (TAVI). However, real-world evidence for TAVI or SAVR outcomes is lacking in Taiwan. This study aimed to compare the clinical outcomes of TAVI and SAVR for treating of AS in Taiwan.

**Materials and methods:**

The National Health Insurance Research Database is a nationally representative cohort that contains detailed registry and claims data from all 23 million residents of Taiwan. This retrospective cohort study used this database to compare patients who underwent SAVR (bioprosthetic valves) or TAVI from 2017 to 2019. Survival outcomes and length of hospital stay (LOS) and intensive care unit (ICU) stay between TAVI and SAVR in the matched cohort. A Cox proportional hazards model was performed to identify the effect of treatment type on survival rates while controlling variables including age, gender, and comorbidities.

**Results:**

We identified 475 and 1605 patients who underwent TAVI and SAVR with a bioprosthetic valve, respectively. Patients who underwent TAVI were older (82.19 vs. 68.75 y/o) and more likely to be female (55.79% vs. 42.31%) compared with patients who underwent SAVR. Propensity score matching (PSM) on age, gender, and Elixhauser Comorbidity Index (ECI) score revealed that 375 patients who underwent TAVI were matched with patients who underwent SAVR. A significant difference was found in survival rates between TAVI and SAVR. The 1-year mortality rate was 11.44% with TAVI and 17.55% with SAVR. Both the mean total LOS (19.86 vs. 28.24 days) and mean ICU stay (6.47 vs. 11.12 days) for patients who underwent TAVI were shorter than those who underwent SAVR.

**Conclusion:**

Patients who had undergone TAVI had better survival outcomes and shorter LOS compared with patients who had undergone SAVR in Taiwan.

## Introduction

AS is a common valvular pathology among heart valve diseases in the age group of over-75 years, with 2% to 4% of this population suffering from the condition [1]. This disease has poor prognoses, with approximately half of the patients with severe symptomatic AS (ssAS) dying within 2 years of symptom onset if left untreated. Aortic valve replacement (AVR) is the only effective treatment option for ssAS [2]. Surgical AVR (SAVR) was the gold standard for treatment for five decades [3]. However, more than one-third of patients with ssAS referred for SAVR are inappropriate candidates due to comorbidities, advanced age, and/or frailty, and a less-invasive procedure may offer an alternative method for their treatment [4, 5].

Transcatheter aortic valve implantation (TAVI) has been recommended as a therapeutic option to AVR for an increasingly wide spectrum of patients with ssAS over the past two decades [6, 7]. TAVI is performed by inserting a bioprosthetic valve through a catheter, which is then implanted within the diseased aortic valve. Increasing numbers of ssAS patients have been treated using this less-invasive technology worldwide since the first TAVI in 2002 [8–10].

Several studies have demonstrated that TAVI was non-inferior or even superior to SAVR across countries in the real-world setting [11–14]. TAVI was introduced in Taiwan in 2012 [15]. However, the comparative study between TAVI and SAVR based on Taiwan’s real-world practice is limited. The present article aimed to compare the survival outcomes of patients who underwent TAVR and those who underwent SAVR in Taiwan using whole-population data. Moreover, the hospital utilization between these two treatments was evaluated.

## Materials and methods

### Data source and patient definition

This retrospective population-based study accessed claim records from the National Health Insurance Research Database (NHIRD) from 2017 to 2019. NHIRD contains details of all the beneficiaries in Taiwan (23,603,121 in 2019). Taiwan’s NHIRD is a public database available through a formal application and approved by the Health and Welfare Data Science Centre of the Ministry of Health and Welfare, Taiwan. This study is based on data from the Health and Welfare Data Science Center in the Ministry of Health and Welfare (H108111). The cause of aortic valve diseases and the inclusion criteria were identified using the International Classification of Diseases (ICD)-10 diagnosis and procedure codes. Patients with two or more outpatient coding or one inpatient coding of ICD-10 I35.0 or I35.2 were identified as AS patients. Patients receiving SAVR were identified by bioprosthetic valve code, porcine valve (FHV02), or durable valve (FHVD1) [16]. Patients who underwent TAVI were identified by procedure code (68040B). We identified 475 patients who underwent TAVI and 1,605 patients who underwent SAVR and received bioprosthetic heart valves.

### Assessment

Baseline characteristics were compared between the TAVI and SAVR groups in the unmatched cohort. Then, a PSM design with one-to-one matching was used. The characteristics used in matching including gender, age(+-2), and ECI score [17]. The matching yielded 376 pairs of well-matched patients.

A Kaplan-Meier survival curve was used for time-to-event analysis in the matched sample, using a stratified log-rank to test the equality of the estimated survival curves for all-cause mortality. The time-to-event stars from the first day of the procedure until death. Additionally, a Cox proportional hazards model, which adjusted for age, gender, and comorbidities, was performed. The difference in comorbidities among treatments was tested by the chi-square test. LOS was measured as the total number of days of hospitalization during SAVR or TAVI.

### Research ethics approval

The study protocol was approved by the MacKay Memorial Hospital Institutional Review Board Taiwan, R.O.C. (Protocol Number: 19MMHIS083e). Informed consent was waived by the ethics committee.

### Data analyzes

Data analyses were performed with SAS 9.4 (SAS Institute Inc., Cary, NC). Variable measures were identified based on the above-described criteria. The categorical variables were described using frequencies or percentages. The mean (Std) was used to describe the continuous variables. The Cox proportional hazards model included the following variables: TAVI, age, gender, congestive heart failure, cardiac arrhythmias, valvular disease, pulmonary circulation disorders, peripheral vascular disorders, hypertension (uncomplicated), hypertension (complicated), paralysis, other neurological disorders, chronic pulmonary disease, diabetes (uncomplicated), diabetes (complicate), hypothyroidism, renal failure, liver disease, peptic ulcer disease (excluding bleeding), metastatic cancer, solid tumor (without metastasis), rheumatoid arthritis/collagen vascular diseases, coagulopathy, weight loss, fluid and electrolyte disorders, blood loss anemia, deficiency anemia, alcohol abuse and depression.

## Results

### Sample description

**Fig 1:**
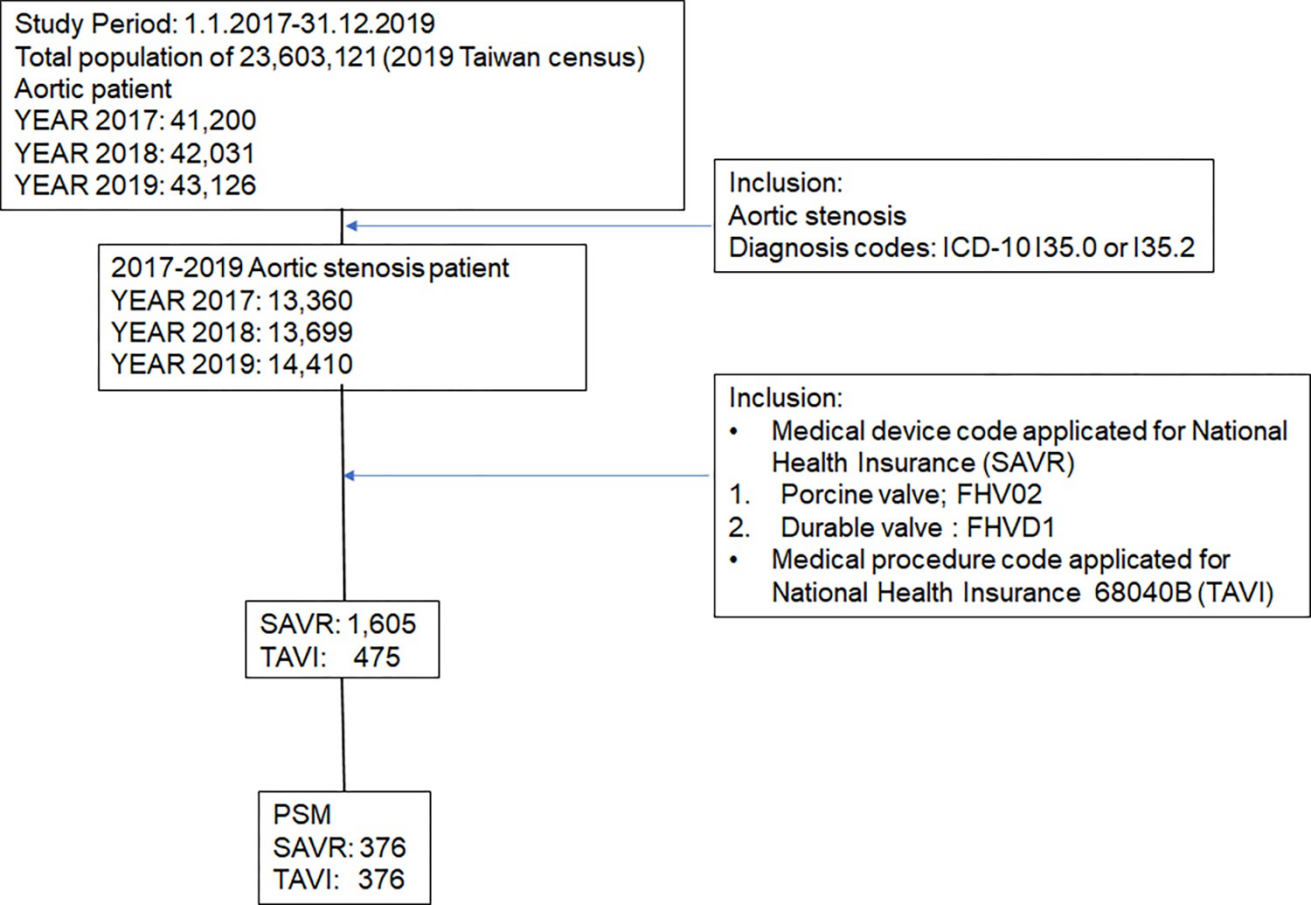
The attrition flowchart of this study.

The cohort was established using the entire population in NHIRD from 2017 to 2019 (Fig 1). Patients receiving TAVI or those with aortic valve disease who had undergone SAVR with a bioprosthetic heart valve replacement were included: 475 TAVI and 1,605 SAVR. Table 1 presents the baseline characteristics. A porcine valve and a durable valve were used in 573 and 1,032 patients among those with AS who received SAVR. Patients who underwent TAVI were older (82.19 vs. 68.75 y/o) and more likely to be female (55.79% vs. 42.31%) compared with those who underwent SAVR. The imbalanced characteristics imbalance was much improved after PSM, with the mean age being 78.64 ± 6.53 and 80.69 ± 7.36 years in the SAVR and TAVI groups, respectively, with the same gender distribution.

**Table 1:** Baseline characteristics before and after propensity score matching between the SAVR group and the TAVI group.

Variable	Unmatched group			Propensity score-matched group		
	SAVR	TAVI	P value	SAVR	TAVI	P value
	(n = 1,605)	(n = 475)		(n = 376)	(n = 376)	
Sex						
Male	926 (57.69%)	210 (44.21%)	< 0.0001	167 (44.41%)	167 (44.41%)	> 0.9999
Female	679 (42.31%)	265 (55.79%)	< 0.0001	209 (55.59%)	209 (55.59%)	> 0.9999
Age						
≤70	889 (55.39%)	33 (6.95%)	< 0.0001	33 (8.78%)	32 (8.51%)	0.8968
>70	716 (44.61%)	442 (93.05%)	< 0.0001	343 (91.22%)	344 (91.49%)	0.8968
Mean (Std)	68.75 (10.39)	82.19 (7.55)	< 0.0001	78.64 (6.53)	80.69 (7.36)	< .0001
ECI score						
Mean (Std)	10.96 (7.09)	12.74 (6.95)	< 0.0001	12.23 (6.31)	12.23 (6.31)	> 0.9999
ECI comorbidity category						
Congestive heart failure	697 (43.43%)	244 (51.37%)	0.0023	197 (52.39%)	185 (49.20%)	0.3815
Cardiac arrhythmias	334 (20.81%)	121 (25.47%)	0.0311	86 (22.87%)	84 (22.34%)	0.8616
Valvular disease	1225 (76.32%)	368 (77.47%)	0.6033	296 (78.72%)	293 (77.93%)	0.7906
Pulmonary circulation disorders	27 (1.68%)	6 (1.26%)	0.5222	5 (1.33%)	≤3 (≤0.80%)	0.4819
Peripheral vascular disorders	113 (7.04)	24 (5.05%)	0.1267	16 (4.26%)	18 (4.79%)	0.7257
Hypertension, uncomplicated	792 (49.35%)	266 (56.00%)	0.0109	233 (61.97%)	205 (54.52%)	0.0386
Hypertension, complicated	498 (31.03%)	194 (40.84%)	0.0001	148 (39.36%)	154 (40.96%)	0.6554
Paralysis	8 (0.5%)	4 (0.84%)	0.3904	0 (0.00%)	≤3 (≤0.80%)	0.1986
Other neurological disorders	1264 (78.75%)	402 (84.63%)	0.0050	323 (85.90%)	328 (87.23%)	0.5930
Chronic pulmonary disease	381 (23.74%)	131 (27.58%)	0.0882	103 (27.39%)	92 (24.47%)	0.3602
Diabetes, uncomplicated	427 (26.60%)	160 (33.68%)	0.0027	118 (31.38%)	131 (34.84%)	0.3139
Diabetes, complicate	317 (19.75%)	108 (22.74%)	0.1566	77 (20.48%)	89 (23.67%)	0.2917
Hypothyroidism	30 (1.87%)	13 (2.74%)	0.2459	6 (1.60%)	11 (2.93%)	0.2269
Renal failure	306 (19.07%)	119 (25.05%)	0.0046	86 (22.87%)	89 (23.67%)	0.7957
Liver disease	73 (4.55%)	15 (3.16%)	0.1885	17 (4.52%)	12 (3.19%)	0.3460
Peptic ulcer disease excluding bleeding	220 (13.71%)	63 (13.26%)	0.8042	60 (15.96%)	51 (13.56%)	0.3553
AIDS/H1V	0 (0.00%)	0 (0.00%)	> 0.9999	0 (0.00%)	0 (0.00%)	> 0.9999
Lymphoma	≤3 (≤0.18%)	4 (0.84%)	0.0483	≤3 (≤0.80%)	≤3 (≤0.80%)	> 0.9999
Metastatic cancer	9 (0.56%)	5 (1.05%)	0.2572	≤3 (≤0.80%)	≤3 (≤0.80%)	> 0.9999
Solid tumor without metastasis	127 (7.91%)	62 (13.05%)	0.0007	32 (8.51%)	45 (11.97%)	0.1195
Rheumatoid arthritis/collagen vascular diseases	71 (4.42%)	21 (4.42%)	0.9981	21 (5.59%)	11 (2.93%)	0.0756
Coagulopathy	14 (0.87%)	6 (1.26%)	0.4458	≤3 (≤0.80%)	4 (1.06%)	0.7051
Obesity	0 (0.00%)	0 (0.00%)	> 0.9999	0 (0.00%)	0 (0.00%)	> 0.9999
Weight loss	11 (0.69%)	6 (1.26%)	0.2264	≤3 (≤0.80%)	4 (1.06%)	0.7051
Fluid and electrolyte disorders	78 (4.86%)	26 (5.47%)	0.5899	15 (3.99%)	18 (4.79%)	0.5938
Blood loss anemia	12 (0.75%)	5 (1.05%)	0.5187	5 (1.33%)	5 (1.33%)	> 0.9999
Deficiency anemia	39 (2.43%)	4 (0.84%)*	0.0415	14 (3.72%)	≤3 (≤0.80%)	0.0142
Alcohol abuse	8 (0.50%)	0 (0.00%)	0.2655	0 (0.00%)	0 (0.00%)	> 0.9999
Drug abuse	≤3 (≤0.18%)	≤3 (0.63%)	0.1353	0 (0.00%)	≤3 (≤0.80%)	0.1974
Psychoses	≤3 (≤0.18%)	≤3 (0.63%)	0.1353	0 (0.00%)	≤3 (≤0.80%)	0.1974
Depression	68 (4.24%)	20 (4.21%)	0.9801	14 (3.72%)	15 (3.99%)	0.8498
Hospital level						
Medical center	1123 (69.97%)	341 (71.79%)	0.4453	258 (68.62%)	265 (7.048%)	0.4769
Regional hospital	455 (28.35%)	134 (28.21%)	0.9531	115 (30.59%)	111 (29.52%)	0.7322
Others	27 (1.68%)	0 (0.00%)	0.0493	3 (0.80%)	0 (0.00%)	0.1967
Length of Stay (day)						
Mean (Std)	24.46 (20.46)	19.96 (24.83)	< 0.0001	28.24 (24.14)	19.86 (24.82)	< 0.0001
ICU stay (day)						
N	1032 (64.30%)	300 (63.16%)	0.6489	247 (65.96%)	243 (64.63%)	0.7589
Mean (Std)	8.13 (12.2)	6.40 (12.02)	< 0.0001	11.12 (16.07)	6.47 (12.38)	< 0.0001
Follow-up time (day)						
Mean (Std)	523.84 (384.1)	473.27 (343.52)	< 0.0001	511.33 (390.39)	470.6 (346.39)	< 0.0001
Mortality						
In-hospital	-	-	-	23 (6.12%)	12 (3.19%)	0.0612
30-day	-	-	-	31 (8.24%)	15 (3.99%)	0.0171

We examined each patient’s medical claims before the heart valve procedure and the result indicated that patients who underwent TAVI had higher rates of these nine underlying diseases before PSM compared to those who underwent SAVR: congestive heart failure, cardiac arrhythmias, hypertension (uncomplicated and complicated), chronic pulmonary disease, diabetes (complicated), liver disease, rheumatoid arthritis, and obesity. The patients in the TAVI group were more likely to be in worse condition than those in the SAVR group (Table 1). The underlying diseases in patients receiving TAVI or SAVR were similar after PSM.

### Hospital resource utilization

The LOS and ICU stay for patients who underwent TAVI or SAVR were measured in this study. The patients who underwent SAVR in unmatched groups exhibited a remarkably longer LOS than those who underwent TAVI (24.46 days vs. 19.96 days, p < 0.001). Similarly, the unmatched ICU stay for patients undergoing SAVR was longer than those who underwent TAVI (8.13 days vs. 6.40 days, p < 0.001). Patients who underwent SAVR also exhibited a remarkably longer LOS than those who underwent TAVI after PSM matching (28.24 days vs. 19.86 days, p < 0.001). The ICU stay for patients undergoingSAVR had a longer ICU stay than those who underwent TAVI (11.12 days vs. 6.47 days, p < 0.001).

### Clinical outcomes of patients who underwent SAVR

**Fig 2:**
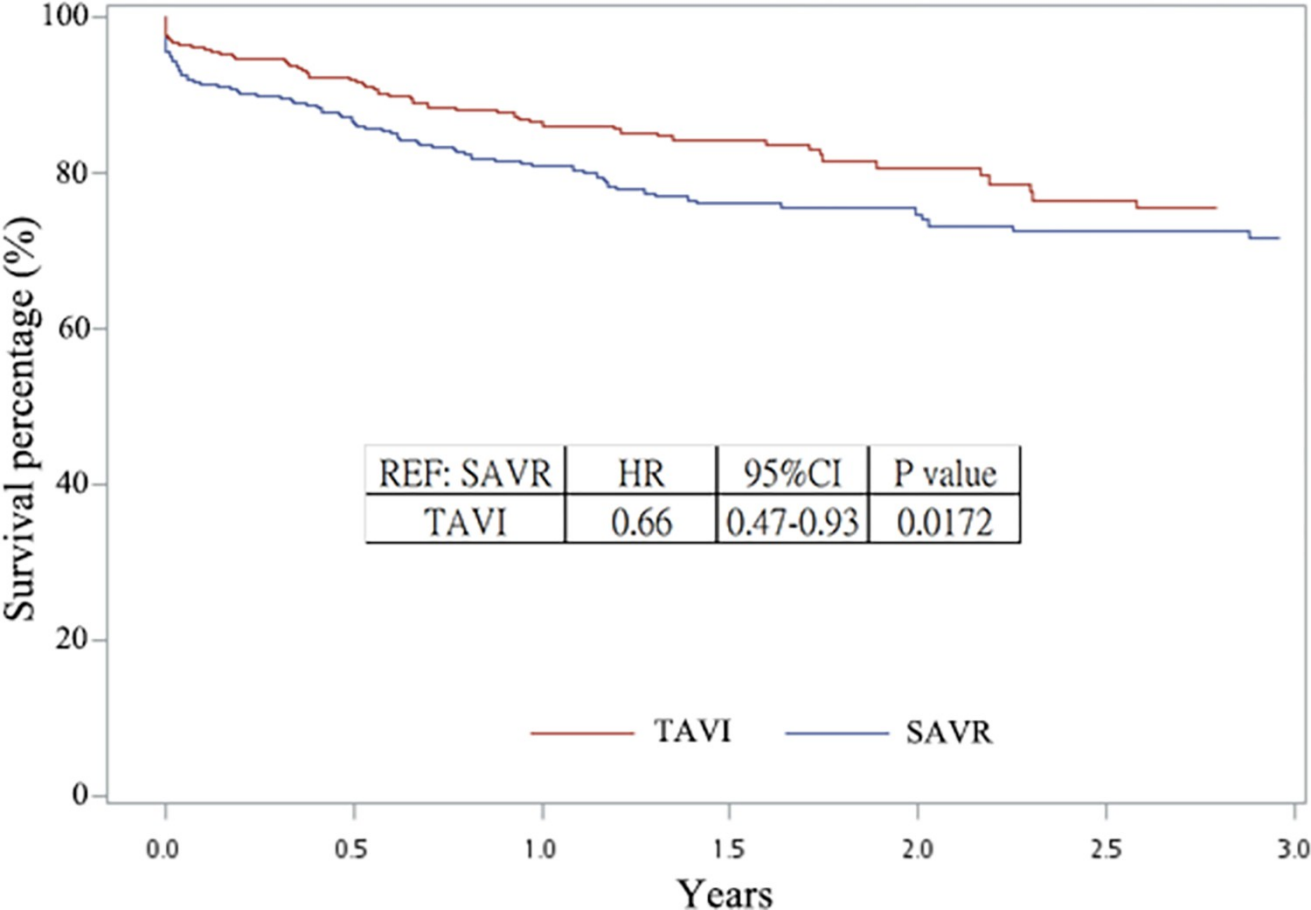
The all-cause mortality of SAVR and TAVI after PSM.

The all-cause mortality was used as the clinical outcome. The mortality rate in patients who underwent TAVI and SAVR after PSM were compared. Patients who received TAVI exhibited lower all-cause mortality rates than those who received SAVR, as shown in Fig 2 (hazard ratio (HR) = 0.66 and p = 0.0172). The 1-year mortality rate was 11.44% with TAVI and 17.55% with SAVR.

### Cox proportional hazards model for patients with TAVI and SAVR

Cox proportional hazards model was used to investigate the clinical manifestations of all patients who had undergone TAVI or SAVR with a bioprosthetic valve. The results revealed that patients with poor outcomes were generally older (1.06, 1.03–1.09) and more likely to have underlying hypothyroidism (1.88, 1.20–2.93), liver disease (2.83, 1.95–4.12), fluid and electrolyte disorders (2.80, 1.48–5.32), and depression (17.07, 6.06–48.08) (Table 2).

**Table 2:** Cox-proportional hazard model for post-procedure mortality.

Variable	HR	95% CI		P value
**TAVI**	0.522	0.361	0.755	0.0006
**Age**	1.059	1.027	1.093	0.0003
**Gender**	0.812	0.582	1.133	0.2213
**Congestive heart failure**	1.001	0.698	1.436	0.9957
**Cardiac arrhythmias**	1.120	0.741	1.694	0.5911
**Valvular disease**	0.708	0.476	1.052	0.0871
**Pulmonary circulation disorders**	1.805	0.436	7.478	0.4154
**Peripheral vascular disorders**	0.952	0.398	2.277	0.9115
**Hypertension, uncomplicated**	0.611	0.430	0.868	0.0060
**Hypertension, complicated**	0.849	0.589	1.225	0.3823
**Paralysis**	1.548	0.189	12.671	0.6836
**Other neurological disorders**	0.692	0.399	1.200	0.1898
**Chronic pulmonary disease**	1.208	0.820	1.780	0.3383
**Diabetes, uncomplicated**	1.064	0.682	1.661	0.7850
**Diabetes, complicate**	1.875	1.199	2.931	0.0059
**Hypothyroidism**	1.257	0.497	3.179	0.6285
**Renal failure**	2.830	1.945	4.116	< .0001
**Liver disease**	1.463	0.653	3.275	0.3549
**Peptic ulcer disease excluding bleeding**	1.324	0.839	2.089	0.2274
**Metastatic cancer**	1.516	0.182	12.624	0.7006
**Solid tumor without metastasis**	5.021	0.575	43.872	0.1445
**Rheumatoid arthritis/collagen vascular diseases**	1.044	0.584	1.866	0.8844
**Coagulopathy**	2.802	1.476	5.321	0.0016
**Weight loss**	0.692	0.081	5.914	0.7363
**Fluid and electrolyte disorders**	17.070	6.061	48.076	< .0001
**Blood loss anemia**	1.453	0.737	2.867	0.2811
**Deficiency anemia**	1.303	0.395	4.299	0.6641
**Alcohol abuse**	1.071	0.420	2.730	0.8855
**Depression**	1.256	0.537	2.935	0.5991

### Complications after surgery

We monitored the complications for 30-day and 90-day spans to understand the postoperative health status among patients who underwent SAVR and TAVI. The 30 day postoperative diagnosis for underlying diseases for both groups was very close to the baseline (Table 3). The 90 day postoperative diagnosis among each treatment group was slightly increased as compared with 30 days.

**Table 3:** Health status after surgery.

Variable	30 days POST-SURGERY		90 days POST-SURGERY	
	SAVR	TAVI	SAVR	TAVI
Congestive heart failure	181 (48.14%)	195 (51.86%)	207 (55.05%)	218 (57.98%)
Cardiac arrhythmias	87 (23.14%)	85 (22.61%)	100 (26.60%)	97 (25.80%)
Valvular disease	303 (80.59%)	304 (80.85%)	332 (88.30%)	335 (89.10%)
Pulmonary circulation disorders	13 (3.46%)	5 (1.33%)	13 (3.46%)	6 (1.60%)
Peripheral vascular disorders	27 (7.18%)	19 (5.05%)	35 (9.31%)	24 (6.38%)
Hypertension, uncomplicated	136 (36.17%)	127 (33.78%)	173 (46.01%)	152 (40.43%)
Hypertension, complicated	118 (31.38%)	136 (36.17%)	140 (37.23%)	158 (42.02%)
Other neurological disorders	220 (58.51%)	239 (63.56%)	273 (72.61%)	286 (76.06%)
Chronic pulmonary disease	46 (12.23%)	40 (10.64%)	72 (19.15%)	57 (15.16%)
Diabetes, uncomplicated	73 (19.41%)	81 (21.54%)	90 (23.94%)	108 (28.72%)
Diabetes, complicate	43 (11.44%)	55 (14.63%)	57 (15.16%)	71 (18.88%)
Hypothyroidism	3 (0.80%)	11 (2.93%)	4 (1.06%)	11 (2.93%)
Renal failure	55 (14.63%)	68 (18.09%)	71 (18.88%)	79 (21.01%)
Liver disease	8 (2.13%)	4 (1.06%)	8 (2.13%)	8 (2.13%)
Peptic ulcer disease excluding bleeding	19 (5.05%)	12 (3.19%)	30 (7.98%)	17 (4.52%)
AIDS/H1V	0 (0.00%)	0 (0.00%)	0 (0.00%)	0 (0.00%)
Solid tumor without metastasis	16 (4.26%)	32 (8.51%)	26 (6.91%)	37 (9.84%)
Rheumatoid arthritis/collagen vascular diseases	7 (1.86%)	4 (1.06%)	7 (1.86%)	4 (1.06%)
Coagulopathy	5 (1.33%)	3 (0.80%)	5 (1.33%)	7 (1.86%)
Obesity	0 (0.00%)	0 (0.00%)	0 (0.00%)	0 (0.00%)
Fluid and electrolyte disorders	7 (1.86%)	12 (3.19%)	14 (3.72%)	18 (4.79%)
Alcohol abuse	0 (0.00%)	0 (0.00%)	0 (0.00%)	0 (0.00%)
Psychoses	0 (0.00%)	0 (0.00%)	0 (0.00%)	0 (0.00%)
Drug abuse	0 (0.00%)	0 (0.00%)	0 (0.00%)	≤3 (≤0.80%)
Weight loss	0 (0.00%)	0 (0.00%)	≤3 (≤0.80%)	≤3 (≤0.80%)
Depression	≤3 (≤0.80%)	3 (0.80%)	≤3 (≤0.80%)	7 (1.86%)
Paralysis	≤3 (≤0.80%)	3 (0.80%)	3 (0.80%)	3 (0.80%)
Lymphoma	≤3 (≤0.80%)	≤3 (≤0.80%)	≤3 (≤0.80%)	≤3 (≤0.80%)
Metastatic cancer	3 (0.80%)	≤3 (≤0.80%)	3 (0.80%)	3 (0.80%)
Blood loss anemia	3 (0.80%)	≤3 (≤0.80%)	7 (1.86%)	4 (1.06%)
Deficiency anemia	4 (1.06%)	≤3 (≤0.80%)	5 (1.33%)	5 (1.33%)

## Discussion

This NHIRD based study mainly revealed a significantly lower risk of all-cause mortality in patients who underwent TAVI than those who underwent SAVR in real-world practice in Taiwan. To our best knowledge, this is the largest real-world outcome study for TAVI in Taiwan in recent years.

This study revealed that most of the patients in the TAVI group were >70 years (N = 442, 93.05%), which indicates that TAVI procedures in Taiwan followed the appropriate guidelines [18]. In contrast, a high number of patients aged ≤70 years received bioprosthetic valve replacement (57.39%). Only 42.31% (N = 679) of patients who underwent SAVR were >70 years old. The percentage of older patients who underwent TAVI was significantly higher than those who underwent SAVR. Age, gender, and comorbidities were most comparable between these groups after matching. The all-cause mortality of SAVR was significantly higher than that of TAVI (p = 0.0172). This may be due to long recovery time needed for open-heart surgeries and complications. Our findings were consistent with several published articles. Two studies reported faster and better left ventricular function recovery and less frequent pulmonary complications after TAVI than after SAVR [19, 20]. These events might potentially increase all-cause mortality associated with SAVR. Another meta-analysis revealed significantly lower 30-day and 1-year all-cause mortality after TAVI than after SAVR [21]. Our results indicate TAVI as a good alternative option for the elderly or for patients who are not suitable for surgery due to some specific condition.

Atrial fibrillation (AF) is one of the most frequent cardiac arrhythmias in the general population associated with high mortality [22, 23]. AF has been associated with several adverse events following SAVR and has been proven as a factor in poor prognoses [24, 25]. Previous studies revealed that TAVI-treated patients have a higher prevalence of preexisting AF compared to patients who underwent SAVR (32.1% vs. 12.8%) and a lower post-procedure rate of AF in TAVI compared to SAVR (6% vs. 33.7%) [26, 27]. However, this study not reveal this result, where the preexisting rates (22.34% vs. 22.87%) and post-procedure rates (30 days after the procedure, 22.61% vs. 23.14% at 30 days; 25.80% vs. 26.30% at 90 days) of cardiac arrhythmias were slightly lower in SAVR. However, cardiac arrhythmias are prevalent comorbidity in patients receiving TAVI, affecting approximately one-quarter of patients in Taiwan, which supports findings in other cohort studies/randomized control trials, although we did not find any significant difference between TAVI and SAVR [28–30]. Hence, cardiac arrhythmias following TAVI can still impact long-term prognoses and treatment outcomes for patients with severe AS.

TAVI procedures have been reimbursed since March 2017, while TAVI medical device reimbursement did not begin until February 2021. The reimbursement guidelines for TAVI are relatively more restrictive than those for other treatments to avoid their overuse and any ensuing financial burden [31]. Patients must meet all of the four essential criteria and at least one of the optional criteria listed below. Essential criteria are: 1) classified as New York Heart Association Function Class II–IV; 2) aortic valve area of <0.8 cm^2^ and <0.6 cm^2^/m^2^, transaortic valve pressure difference≧40 mmHg, or aortic valve blood flow velocity≧4.0 m/sec; 3) at least two cardiac surgery specialists must judge that SAVR cannot be used for AVR or the risk posed by surgery is too high; and 4) the patient life expectancy after TAVI is more than 1 year. Concurrently, at least one of the following three optional criteria must be met: 1) STS Score >10% or Logistic EuroSCORE I > 20%; 2) >80 years old; or 3) previous heart surgery (coronary artery bypass and heart valve surgery) or mediastinal radiation therapy, severe aortic calcification (porcelain aorta), ineligible for open-heart surgery due to thoracic cauterization sequelae, severe connective tissue disease leading to inoperability, liver cirrhosis (Child class A or B) with pulmonary insufficiency (FEV < 1 liter). TAVI medical devices were not eligible for reimbursement in this study period (2017–2019), and patients needed to cover the costs by themselves or be privately insured for TAVI medical devices.

The analyzes of the NHIRD data have provided several benefits, but such data still have limitations due to the nature of the NHIRD design. First, lacking information on self-payment for medications, laboratory data, and patient information (height, weight, etc.) due to NHIRD was designed as a claim database. Second, the severity of the disease cannot be measured. Third, this study design was strictly based on the ICD-10-CM, ICD-10-OP systems, thus coding errors, misclassifications, or differences between hospitals and physicians can affect these results, making the proportions of our subjects incorrect. Finally, TAVI was not reimbursed in Taiwan until March 2017. The National Health Insurance Administration needs almost a year to systematically list annual data and release them. Hence, our study cannot include more patients who underwent TAVI for long-term observation until more data are updated.

## Conclusion

This study revealed that patients in the TAVI group were older and more likely to be in worse condition than patients receiving SAVR. The all-cause mortality of patients who underwent TAVI was lower than those who underwent SAVR after PSM by age, gender, and ECI score. Patients who underwent TAVI had significantly shorter LOS and ICU stays than those who underwent SAVR. These findings indicate statistically significant differences in survival rates in TAVI compared to SAVR in these older patients with severe AS. Further studies over a long-term are required to understand the clinical and economic outcomes of TAVI.
